# Cortical alpha oscillations in cochlear implant users reflect subjective listening effort during speech-in-noise perception

**DOI:** 10.1371/journal.pone.0254162

**Published:** 2021-07-09

**Authors:** Brandon T. Paul, Joseph Chen, Trung Le, Vincent Lin, Andrew Dimitrijevic

**Affiliations:** 1 Evaluative Clinical Sciences Platform, Sunnybrook Research Institute, Toronto, Ontario, Canada; 2 Otolaryngology—Head and Neck Surgery, Sunnybrook Health Sciences Centre, Toronto, Ontario, Canada; 3 Faculty of Medicine, Otolaryngology—Head and Neck Surgery, University of Toronto, Toronto, Ontario, Canada; Medical University Hannover; Cluster of Excellence Hearing4all, GERMANY

## Abstract

Listening to speech in noise is effortful for individuals with hearing loss, even if they have received a hearing prosthesis such as a hearing aid or cochlear implant (CI). At present, little is known about the neural functions that support listening effort. One form of neural activity that has been suggested to reflect listening effort is the power of 8–12 Hz (alpha) oscillations measured by electroencephalography (EEG). Alpha power in two cortical regions has been associated with effortful listening—left inferior frontal gyrus (IFG), and parietal cortex—but these relationships have not been examined in the same listeners. Further, there are few studies available investigating neural correlates of effort in the individuals with cochlear implants. Here we tested 16 CI users in a novel effort-focused speech-in-noise listening paradigm, and confirm a relationship between alpha power and self-reported effort ratings in parietal regions, but not left IFG. The parietal relationship was not linear but quadratic, with alpha power comparatively lower when effort ratings were at the top and bottom of the effort scale, and higher when effort ratings were in the middle of the scale. Results are discussed in terms of cognitive systems that are engaged in difficult listening situations, and the implication for clinical translation.

## Introduction

Individuals who are listening to speech in noisy places, such as crowded restaurants or workplace settings, often report that it takes more effort to understand what they are hearing. Several factors make listening under these conditions difficult compared to quiet, such as fewer acoustic details available to the listener, and the distracting influence of competing sounds [1]. The effort required to listen to speech in noise is even greater for those with hearing loss, even after receiving properly fitted hearing aids [2,3]. These findings suggest that the demand on a listener with hearing loss is still high despite the increase in audibility and speech understanding. As a result, the concept of “listening effort” has received mainstream focus as a fundamental, but still poorly understood, part of human communication [4–8]. The goals for studying listening effort are to understand its neural basis, and to develop reliable measurement tools for use in clinical populations who find everyday listening chronically effortful [4,5].

A recent consensus paper and conceptual model outlined by Pichora-Fuller et al. [5] defined listening effort as “the deliberate allocation of mental resources to overcome obstacles in goal pursuit when carrying out a [listening] task.” Obstacles include cognitive factors specific to the listener (e.g., linguistic ability, language familiarity, and memory capacity) or type and degree of acoustic challenge (e.g., hearing loss, level of noise, and competing signals). Motivation, as related to listener arousal and fatigue, is also an important determinant of effort [9]. Mental resources in this model refer to a limited reserve of cognitive ability known to be involved in speech listening. For instance, degraded speech or environments with acoustic noise increase the load on working memory [10], engage attention systems more strongly [11], and increase the time needed for perceptual processing [12]. For those with hearing loss, prolonged effort drains the limited amount of resources available, and strain on these systems can generate fatigue from listening overexertion [13] and frustration from having to ask others to constantly repeat themselves. Notably, these prolonged listening challenges can lead to social withdrawal and chronic health issues associated with hearing loss, including depression [4,14–16].

Presently there is no agreement on how to measure listening effort, and little is known about its neural basis. A recent review from Francis and Love [8] suggested that listening effort is a multidimensional and dynamic process, and likely involves several overlapping systems implicated in cognition, affect, executive function, language, and sensory processing. Several types of listening effort measurements have been proposed, aimed at capturing the function of one or more of these systems. However, many studies use methods that only indirectly reflect effort-related brain activity. Examples include behavioral reaction times (reviewed in [4]) or performance on dual-task experiments [17], and by indirect physiological measures such as arousal-mediated pupillometry [18], skin conductance [19], and modulations of cardiographic activity [20].

Two candidate listening effort measures of direct relevance to clinical applications are self-report scales and neuroimaging. Self-report measures, such as the subjective rating of effort a listener feels they are giving, capture the primary complaint that many individuals bring to the clinic, and thus convey important information during the course of hearing rehabilitation (see [21]). Examples of self-report tools are simple visual analogue or numerical rating scales [22], or more elaborate questionnaires such as the NASA Task Load Index (NASA-TLX, [23]). Self-reported effort measured on subjective rating scales may be akin to what has been recently characterized as “experienced effort” [24]. Experienced listening effort can be described as the aspect of listening effort that is explicit to a listener (or consciously perceived) following the application of neurocognitive resources under a demanding listening task; or perhaps, the degree of effort that a listener believes they exerted. Experienced effort may be compared to “invested effort,” which can be characterized as the neural resources that were actually applied (and not necessarily explicit to the listener) in demanding listening tasks [24].

On the other hand, noninvasive neural measurements are attractive because they directly concerned with the brain activity underlying listening effort. For example, the power of neural oscillations in the 8–12 Hz band (i.e., alpha range) measured by magnetoencephalography or electroencephalography (M/EEG) has been suggested to index listening effort, because activity in this frequency range has been observed to be modulated by attention and memory load when listeners are asked to attend to speech that is presented in noise or has been spectrally degraded [25–28]. Relatively increased alpha power in these tasks is hypothesized to arise from the synchronization of inhibitory circuits that suppress neural activity in task-irrelevant brain areas (e.g., visual or sensory areas) or the representation of task-distracting acoustic features, thereby protecting speech signals that were targeted and encoded. In contrast, decreased alpha activity signals less inhibition, and thus more excitation, to best represent the sensory signals that were encoded [29–31].

Both increases and decreases of alpha power (relative to baseline) are known to co-occur in separate brain areas and may reflect different aspects of effortful listening. When compared to alpha power during passive listening, Dimitrijevic et al. [28] found that active (task-driven) listening increases alpha power (event-related synchronization, ERS) in parietal brain areas but decreases (event-related desynchronization, ERD) alpha power in temporal sources, compared to passive listening. Notably, only the temporal alpha power decrease was correlated to performance on a speech-in-noise listening task. Thus, alpha power could reflect listening effort level by tracking how resources are applied when balancing the excitatory or inhibitory brain processes listeners use to understand speech, such as parsing speech signals from noise, suppressing irrelevant information, accessing linguistic information, or storing items in memory [7,8].

Currently, it is not clear if oscillatory alpha power reflects the amount of effort a listener feels they are exerting. Manipulations of listening difficulty by way of degrading speech or adding background noise appear to modulate alpha power measured from parietal scalp areas, and for this reason, parietal alpha power has received attention as a possible neural correlate of listening effort [25–28,32–34]. However, the relationship of parietal alpha oscillations to self-reported listening effort ratings *per se* is largely unclear, as their relationship is typically indirectly inferred. For instance, Decruy et al. [34] and McMahon et al. [32] both found that alpha power in parietal areas changes as a function of task difficulty (changing speech-to-noise ratios (SNRs) or level of speech understanding in noise), but these relationships were different from those between self-reported effort ratings and task difficulty changes. In other words, the suggestion is that self-reported effort does not relate to speech-in-noise listening or understanding in the same way as parietal alpha power, and may reflect different subcomponents of effortful listening [34].

Recently, Dimitrijevic et al. [35] investigated the neural correlates of effort in cochlear implant users during speech-in-noise listening by using EEG source analysis. Using a between-subjects design, they found that individual differences in self-reported listening effort measured by the NASA-TLX positively correlated with alpha power in left frontal brain areas. The highest correlation localized to left inferior frontal gyrus (IFG), a region associated with language processing [36] that is well established to be involved in difficult listening tasks [37]. The linear correlation suggested that more effort was associated with relatively increased alpha power and less effort was associated with decreased alpha power. In contrast, correlations of effort ratings to parietal alpha power did not reach significance. The interpretation was that self-reported listening effort for speech relates to modulation of brain oscillations in canonical language areas.

The objective of the present study was to reformat the experimental design of Dimitrijevic et al. [35] to examine if *within-subject* variability in cortical alpha power is related to subjectively related listening effort, as opposed to *between* subject differences reported previously. To achieve this, we manipulated effort for each participant in an innovative methodology by presenting speech material across range of SNRs, rather than focusing on inter-individual differences at a fixed listening or performance level. After each trial, participants rated the effort level that they exerted on a 1–10 scale. The hypotheses tested were that listening effort explains variability in alpha power in (1) parietal sensor areas and (2) left IFG. To do this, we performed separate analyses of sensor and source data in order to match analysis conventions that were used in past studies so that reported data can be compared to those in this experiment. Here, we are not testing if alpha power in left IFG is different from alpha power in parietal sensors, with respect to their relationship to subjective listening effort ratings.

As reported in Dimitrijevic et al. [35], we tested a group of individuals who received a cochlear implant (CI) to treat their severe hearing loss. A CI is a prosthesis that is surgically implanted into the cochlea of the inner ear. Simply, the CI transduces sound pressure waves into electrical pulses that are delivered directly to the auditory nerve inside the cochlea. Although speech is generally perceived and understood to a successful degree, the manner of stimulation and encoded features of speech are fundamentally different from hearing aid users and unaided listeners, and the number of frequency “channels” are limited in CIs compared to aided or unaided listeners. Thus, for CI users, speech is spectrally degraded and is conveyed through different patterns of activation. CI users commonly report that listening to speech is more effortful due to the degradation, even more so in noise, and thus this population is ideal for measuring brain correlates of listening effort [38].

## Methods and materials

### Participants

Sixteen CI users were recruited from the patient population of Sunnybrook Health Sciences Centre, Department of Otolaryngology, and participated in the study. Demographic characteristics and information about their CI use are provided in Table 1. Participant ages ranged from 23 to 75 (M = 59.2, SD = 14.2), and included 7 males and 9 females. All participants used MED-EL (Innsbruck, Austria) implants and processors. The majority of participants were unilateral CI users (N = 13) who in everyday settings use a hearing aid in the contralateral ear (7) or are unaided (6). During the study, only the CI was used. The remaining three were bilateral CI users who received their devices sequentially (delays between implants were 5 months, 2 years, and 3 years). In these cases, only the CI that was implanted first was used during testing. The participants used the CI that was tested for an average of 4 years and 11 months (range: 1 yr 2 mo– 8 yr 1 mo). Clinical speech scores were assessed by measuring consonant-nucleus-consonant (CNC) word recognition in quiet [39,40], a standard measure of speech ability in CI users, and scores for each participant are also listed in Table 1. CNC word scores were measured in the clinic between three months to one year after CI activation. CNC word scores are noticeably lower for Participant #1 compared to the rest of the sample. We note that no results in this report change if this individual is excluded from analysis.

**Table 1:** Participant demographics.

ID	Age	Sex	Etiology	CI side	Electrode Length (mm)	Duration of CI Use	Non-tested Side	CNC Word Recognition in Quiet (%)	Duration of deafness (yrs)
1	23	M	Unknown	R	31	4 yr 2 mo	Hearing Aid	2	Unknown
2	44	F	Sudden deafness	L	31	1 yr 4 mo	None	56	27
3	48	F	Progressive	R	28	5 yr 11 mo	CI	84	10+
4	50	F	Progressive	L	31	8 yr 0 mo	None	44	15
5	51	F	Congenital	R	31	6 yr 10 mo	Hearing Aid	N.A.	51
6	53	M	Sudden deafness	L	31	6 yr 11 mo	None	44	21
7	56	M	Progressive	L	31	2 yrs 2 mo	Hearing Aid	16	56
8	57	F	Unknown	L	31	2 yr 0 mo	None	80	Unknown
9	62	M	Congenital	L	28	2 yr 11 mo	CI	38	62
10	68	F	Progressive	L	28	2 yr 2 mo	Hearing Aid	84	19
11	71	F	Unknown	R	28	1 yr 2 mo	None	80	Unknown
12	71	M	Sudden deafness	L	31	6 yr 5 mo	Hearing Aid	68	30
13	71	M	Progressive	R	24	5 yr 2 mo	CI	72	Unknown
14	73	M	Progressive	L	31	8 yr 1 mo	None	38	Unknown
15	74	F	Progressive	R	31	5 yr 9 mo	Hearing Aid	18	Unknown
16	75	F	Progressive	R	28	5 yr 9 mo	Hearing Aid	30	Unknown

M = male; F = female; CI = cochlear implant; L = left, R = right; yr = year; mo = month, N.A. = not available.

Exclusion criteria were non-fluency in the English language, or self-reported neurological or mental health issues. No recruited participants were excluded on these bases. All participants provided written and informed consent for the study procedures, which were conducted in accordance with Research Ethics Board (REB) at Sunnybrook Health Sciences Centre. Approved protocol and were in agreement with the Declaration of Helsinki. Participants were monetarily compensated for their participation, and were provided full reimbursement for parking at the hospital campus.

### Stimuli and materials

**Stimuli and testing environment.** The primary experimental paradigm used in this study was the digit triplet test, wherein individuals must listen to and report a series of three spoken digits [41] that has been used in previous EEG studies [28,35]. The stimuli were a series of three digits (numbers) spoken by a female talker of standard American English. Digits were recorded in Computerized Speech Lab hardware and software (Kay Elemetrics). Included in the stimulus set were nine monosyllabic digits 0 through 9, with “0” pronounced as “Oh” (/oω/), and excluding the disyllabic “7”. During stimulus recording, digits were recorded as triplets in order to preserve the natural prosody of the spoken series, but each digit was reviewed by experimenters to choose exemplars that excluded prosodic irregularities, hesitations, and acoustic distortion. The final series of selected digits ranged from 434 to 672 milliseconds (ms) in duration (SD = 57 ms), and silence was appended to the end of each digital file to equate their presentation to 695 ms. The final stimulus set was 27 unique digital audio files (nine digits for each of the three positions). The amplitude of the digits was adjusted by a scale factor to equalize the root mean squared (RMS) amplitude of all digits.

**Fig 1:**
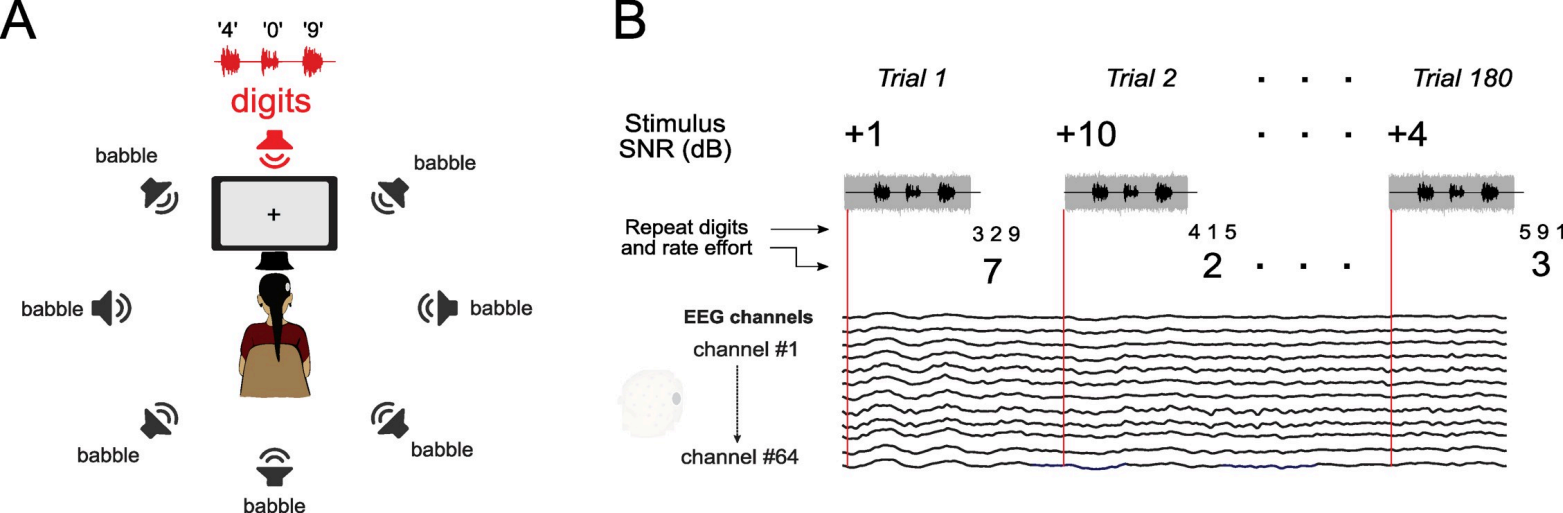
Experimental design. A) Participants were presented with triplets of digits spoken by a female talker at the front-facing speaker, while multi-talker babble noise of varying SNR was presented in the seven peripheral speakers. B) During EEG recording, trials of the digit triplet test were presented, with SNR varying on each trial. After the stimulus presentation ended, participants reported the digits they perceived as well as their listening effort rating on a 1–10 verbal report scale.

During presentation, digits occurred at an onset-to-onset interval of 1195 ms. The digits in each trial were determined pseudo-randomly, where no repeating digits were allowed, nor were digit presentations of ascending or descending order. The digits were presented in the order in which they were recorded in the triplet series. Shown in Fig 1A, all stimuli were presented through a circular ring array of eight speakers, with the participant positioned at the center. Each speaker was 80 cm away from the center of the ring, with respective speaker positions at 0, +/− 45, +/− 90, +/− 135, and 180 degrees. The center of each speaker’s cone was positioned 100 cm from the floor. Digits were always presented through the center speaker at 0 degrees and measured at 65 dBA at the center of the array (in the same horizontal plane as the speaker cone center). In each of the remaining seven speakers, four-talker babble noise taken from the QuickSIN program [42] was presented, and noise levels were varied randomly during the study (see below). The four-talker babble noise files were presented in each speaker at a random phase so that no two speakers had aligning presentation of noise. Digits and babble noise files were processed in MATLAB 2009b (The MathWorks, Natick, MA), and their presentation was controlled by a Tucker Davis Technologies (TDT) RX8 Processor.

For all phases of the study, the trial structure of the digits-in-noise test proceeded as follows (Fig 1B): First, the multi-talker babble noise commenced in the peripheral speakers for three seconds. Then, digits were presented from the center speaker, and the noise ceased 500 ms after the offset of the final digit. Participants then verbally reported the three digits they perceived, and were instructed to do so only when the noise had ceased.

#### Self-reported listening effort

After verbally reporting the digits that they perceived, participants also verbally indicated their listening effort on a 1–10 scale. Listening effort was explicitly and verbally defined to each participant by the experimenter, and was also printed on a posted paper sheet as *Listening Effort*: *the mental demand required to understand the spoken numbers*, with “1” anchored as *least effort possible* and “10” as *most effort possible*. If the participants did not perceive any digits or felt they did not give effort owing to lapses in attention, they were instructed to report “not applicable.” The advantage of a simplified scale to measure effort was the speed and ease at which listeners could respond after each trial. When describing instructions to participants, care was taken by the experimenters to describe the difference between the “effort” and “perceived difficulty.” Participants were specifically instructed to not report how “hard” the trial appeared to be due to the noise level, only report the effort they felt they were giving when listening to the spoken digits. These instructions were given to participants prior to the pre-experiment task and main experiment, which are described in the following sections.

### Procedures

#### Pre-experiment task

Our goal was to measure neural activity that encompassed the range of a CI users’ self-reported listening effort. To achieve this, we conducted a pre-experiment behavioral task to determine the signal-to-noise ratios (SNRs) of the digits-in-noise test that corresponded to the participants’ lowest and highest amount of perceived effort, as defined by the 1 to 10 scale. Starting at a level of +5 dB SNR, a psychophysical staircase procedure was used to increase the SNR of the digit triplet test in 2 dB steps until the participant reported that their effort was at a level of “2” on the 1 to 10 scale. After, the SNR was then decreased from +5 dB SNR in 2 dB steps until the effort rating was reported as a “9”. This protocol was repeated 3 times. The median of the SNR values at each of the “end points” (i.e., effort of 2 and effort of 9) were computed, and the resulting range of SNRs was used during the main experimental task. The endpoints of 1 and 10 were not used in the staircase procedure to avoid exceeding the floor and ceiling of the scale.

#### Main study

During the main experiment, the SNR of the digit triplet test was used to manipulate the participants’ listening effort. However, in order to constrain the experimental SNR levels across participants, SNRs were selected from predefined values starting at −11 dB and increasing in 3 dB steps (e.g., −8, −5, −2, +1, +4). For each participant, the closest value on this array to the effort end points was obtained, and between these values, six equally spaced SNRs were chosen. For example, if a participant’s effort endpoints 0 dB and 17 dB SNR (resulting in effort ratings of 9 and 2), the experimental SNRs were set as 1, 4, 7, 10, 13, and 16 dB SNR. This constraint afforded the ability to, in future analyses, look at individual differences in neural activity across all individuals in the study at fixed SNRs, despite the fact that the range of SNRs for each participant varied owing to their specific effort ability. Two participants, however, had SNR ranges that were too small to fit six SNRs spaced by 3 dB. As a result, these individuals were tested at four SNRs, and both had identical values of 1, 4, 7, and 10 dB SNR.

The main experimental procedure is shown in Fig 1B. On each trial, the SNR of each stimulus was randomly determined. As a consequence of the stimulus design, the sound intensity of the noise onset could possibly cue the participant if the trial was going to be a high or low SNR. To avoid this circumstance, we randomly roved the overall stimulus level (signal plus noise) on each trial such that it was attenuated between 0 and −6 dB. Trials of the digit triplet test were presented in six blocks of 30 trials, totaling 180 trials. Across the study, there were 30 trials for each SNR. Depending on the speed of participants’ response, blocks lasted from five to nine minutes. Between blocks, participants were able to rest as needed. The total experimental session, including set-up, lasted three hours on average.

### EEG analysis

#### Recording and preprocessing

The EEG was recorded from 64 equidistant sensors on an ActiCAP (BrainProducts, Gilching, Germany) cap, covering a comparatively larger area than a 10–20 system, to improve source localization estimates [28,35].The EEG was sampled continuously at 2000 Hz using a NeuroScan SynAmps II amplifier (Compumedics Ltd, Victoria, Australia). EEG signals were referenced to the vertex electrode, with a ground placed on the midline halfway between the nasion and vertex. Following the study, participants’ electrode positions were digitized to a three-dimensional map using a Polhemus Patriot (Polhemus, Colchester, VT, USA). Sensors that covered, or were in close proximity to, the magnet and coil of the CI (between 1 and 4 sensors over temporal regions) were not recorded during the session.

Offline, in BrainVision Analyzer software (Brain Products, Gilching, Germany), EEG data were filtered from 0.1 to 40 Hz using a 2nd-order Butterworth filter and downsampled to 250 Hz. Trials in which there was large artifactual noise were marked, and continuous data ignoring these segments were subjected to independent components analysis (ICA). Visual inspection was used to identify and correct for spatiotemporal patterns of biological contaminants in the EEG signal expressed as independent components, including eye blinks, horizontal eye movement, heartbeat artifacts, and other myogenic artifacts. Between two and seven components were removed for each participant. Following ICA correction, noisy channels and those not recorded over the CI magnet and coil, were replaced with derived estimates from neighboring sensors using spline interpolation.

EEG data were subsequently exported to MATLAB 2018a, and imported using the *Fieldtrip* software [43]. Continuous data were first epoched from −1 to 7 seconds relative to the start of the background noise (0 seconds). Trials and channels with RMS amplitudes that exceeded two standard deviations from the mean were marked. After visual inspection, trial epochs with transient artifacts were removed, on average ~18% of trials across participants. Noisy channels were discarded and replaced with derived estimates from neighboring sensors using spline interpolation. Between 0 and 3 channels were interpolated across all participants.

#### CI artifact suppression

ICA methods have been widely used to suppress the CI artifact and EEG recordings [44–46], and here we used the second-order blind identification (SOBI) algorithm borrowed from EEGLAB functions [47] for CI artifact suppression. Our group has successfully used this procedure to attenuate artifacts in prior studies on CI users [48]. SOBI identifies spatiotemporal EEG patterns based on second-order statistics to separate temporally correlated signals [49]. SOBI was applied on EEG data epochs that were appended into a continuous voltage time series. For each participant, the topographical IC weighting maps were visually inspected alongside time series of IC activations. Those components matching the spatial location of the CI, and with activation time series overlapping with the time at which sound was presented, were set to 0 before reconstruction of the continuous EEG. Between 0 and 2 components were removed per participant.

#### Analysis of alpha power at parietal sensors

Following CI artifact suppression, EEG data were re-referenced to the average of all channels, and single trials were baseline corrected to the −1 to 0 pre-stimulus period. The power of neural oscillations for single trials in each participant was calculated using the multi-taper convolution method in *Fieldtrip*. Multi-taper convolution uses Hanning tapers applied to a sliding-window fast Fourier transform to restrict temporal spread within the window. This method was used to construct time-frequency representations across the entire trial in .048-second steps and in 1 Hz steps from 2–30 Hz. Estimated power values for each frequency bin across the entire trial were then normalized by calculating the post-stimulus period as a decibel change from baseline [10*log_10_ (post/pre)]. After construction of the time-frequency representations, alpha power values for single trials were averaged across the 8–12 Hz frequency band and across the 3–6 second time window. Seven parietal sensors for analysis were chosen after creating a grand average of all trials and participants and examining the scalp topography (shown later in results in Fig 4A). The average of these sensors was taken for each trial. The end result was a single alpha power value for each participant and each trial, which was subjected to statistical analysis.

#### Analysis of alpha power estimated from left inferior frontal gyrus

Each participant’s surface electrode positions were used alongside a standard Montreal Neurological Institute (MNI) template to calculate lead fields with a grid resolution of 15 mm. Using these lead fields, A linearly constrained minimum variance (LCMV) beamformer was applied to the trial-averaged, wideband voltage time series for each individual to construct a set of common spatial filters across the entire brain. Beamformers were computed using 5% regularization, and retained only the largest dipole directions. Spatial filter weights for the left IFG were taken based on the location for the maximum correlation reported in Dimitrijevic et al. [35], which peaked at Talairach coordinates [−39, 11, 10]. The spatial filter for this location was applied to single trial data for each participant. Time-frequency analysis, baseline normalization, and alpha power averaging were applied to spatially filtered single trials using identical steps taken for parietal sensors reported above. Thus, for each subject, a single alpha power value for each trial was retained, and submitted to statistical analysis.

### Statistical analysis

All statistical analyses were performed in R (R Core Team, 2019). The alpha criterion for Type I error for all tests was set at 0.05. For all analyses, trials on which participants reported “not applicable” were removed before analysis.

Behavioural data were analyzed in two ways. The first set of analyses examined interindividual relationships between subject-average effort ratings, task performance (a correct response was a trial in which participants reported all three digits in order correctly), SNR ranges, and SNR maxima and minima, using Spearman rank correlations. The second analysis examined single-trial behavioural performance (1 = all digits correctly reported; 0 = not all digits correctly reported) by fitting a logistic mixed-effects model (*glmer*) with the predictors of listening effort rating, trial SNR. Predictors were treated as continuous variables and were z-scored prior to modeling. The random effects structure included by-subject random intercepts, and correlated by-subject random slopes for both effort and SNR [50]. Significance of fixed effects terms was assessed by iteratively dropping each fixed effect, and identifying differences between the full model and reduced model using likelihood ratio tests. Fixed effects terms are characterized by their odds ratio (OR), standard error (SE), and its slope coefficients are expressed as significantly different from zero using Wald tests.

Single-trial neural data (both sensor and source level) were similarly analyzed by fitting a linear mixed-effects model (*lmer*) to predict alpha power from fixed effects effort ratings and the trial SNR. Alpha power values, SNR values, and effort ratings were treated as continuous variables and were z-scored prior to modeling, and therefore model values are reported as standardized slope coefficients and standardized errors to aid comparison between sensor and source data. Quadratic terms for effort and SNR were also included alongside linear terms, as several studies have shown nonlinear relationships between physiological measures and conditions of effortful listening tasks [32,34,51,52]. By-subject random intercepts were included as a random effect to account for subject-level differences. Random slopes and quadratics for fixed effects terms were initially included to generate a maximal random effects structure [50], but resulted in singular fits which indicate that the random effects structure is too complex to be estimated and is potentially overfit. Random effects structures were iteratively reduced in an attempt to specify a model that converged or was not singularly fit; however, this was not achieved for any random effects structure that included a random slope. Thus, final reported models only include random by-subject intercepts. We note here that results did not qualitatively change (p’s < 0.05) when examining model terms that included the full random effects structure although the fit was singular, but are not reported due to poorly estimated random effects structures. The significance of fixed effects in the final linear models for neural data was determined using an analysis of variance (ANOVA), with degrees of freedom adjusted by the Satterthwaite method [53]. For all mixed effects models, full tables for slope coefficients, standard errors, test statistics, and p values for all fixed effects terms are found in S1 Table. By virtue of the task design, effort and SNR are expected to correlate, which might introduce multicollinearity in the mixed effects modeling. Variance inflation factors (VIFs) were computed for each fixed effects term in the model, and all values were below 2.4. VIFs at and above 5 indicate potentially problematic multicollinearity.

## Results

### Behavioral performance

A summary of individual behavioral performance is given in Table 2. The range of SNRs used in the task varied considerably, with the lowest range encompassing −5 to 10 dB SNR, and the highest range spanned from 7 to 37 dB SNR. Performance on the task was well above chance for all participants, and averaged 71.0%. The grand average of effort ratings on the 1–10 scale was 5.9. No correlations were found between average task performance, average effort rating, or SNR medians or ranges (all uncorrected ps > 0.19). These results suggest that subject-specific task parameters did not have an observable effect on the average level of effort and individual differences in speech-in-noise performance.

**Table 2:** Digits-in-noise task parameters and average performance for each participant.

Participant	SNR Min (dB)	SNR Max (dB)	Task Performance (%)	Mean Effort Rating (out of 10)
1	4	19	15	4.4
2	−5	10	57	7
3	4	19	95	5.4
4	4	19	89	5.8
5	7	22	98	4.8
6	7	35	67	3.5
7	−2	13	66	5.3
8	−5	10	97	6.9
9	1	10	41	7.7
10	−2	13	81	4.4
11	1	16	78	9.2
12	1	10	75	4.3
13	1	16	72	7.1
14	1	16	64	6.5
15	7	37	66	5.8
16	1	16	73	8

SNR = signal to noise ratio.

**Fig 2:**
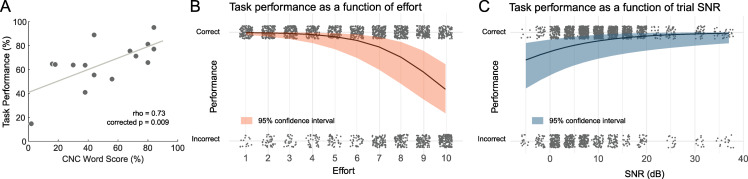
Behavioural data. A) Correlation between CNC Word Score (a clinical test of word recognition) and performance on the task. The line represents the least squares fit. “Rho” indicates Spearman’s correlation coefficient. B) Performance predictions from the logistic mixed effects model plotted against individual trials (grey dots). Note that dots are randomly jittered around each effort level and performance outcome for visibility. The fitted line represents model predictions of performance from subjective effort, with the orange shaded region bounding the 95% confidence interval. C) The same as panel B, but plotting performance as a function of SNR.

Correlations between clinical speech scores (CNC word recognition) were also compared to task performance and task parameters. No significant relationships were found between CNC word recognition and average effort ratings or SNR characteristics. However, the correlation between CNC word recognition and task performance was significant (Spearman’s rho = 0.73; p = 0.009, FDR corrected) and suggested a positive relationship between the two performance scores (Fig 2A). Thus, the CI users’ performance on the experimental task was similar to outcomes measured through a validated clinical speech test.

**Fig 3:**
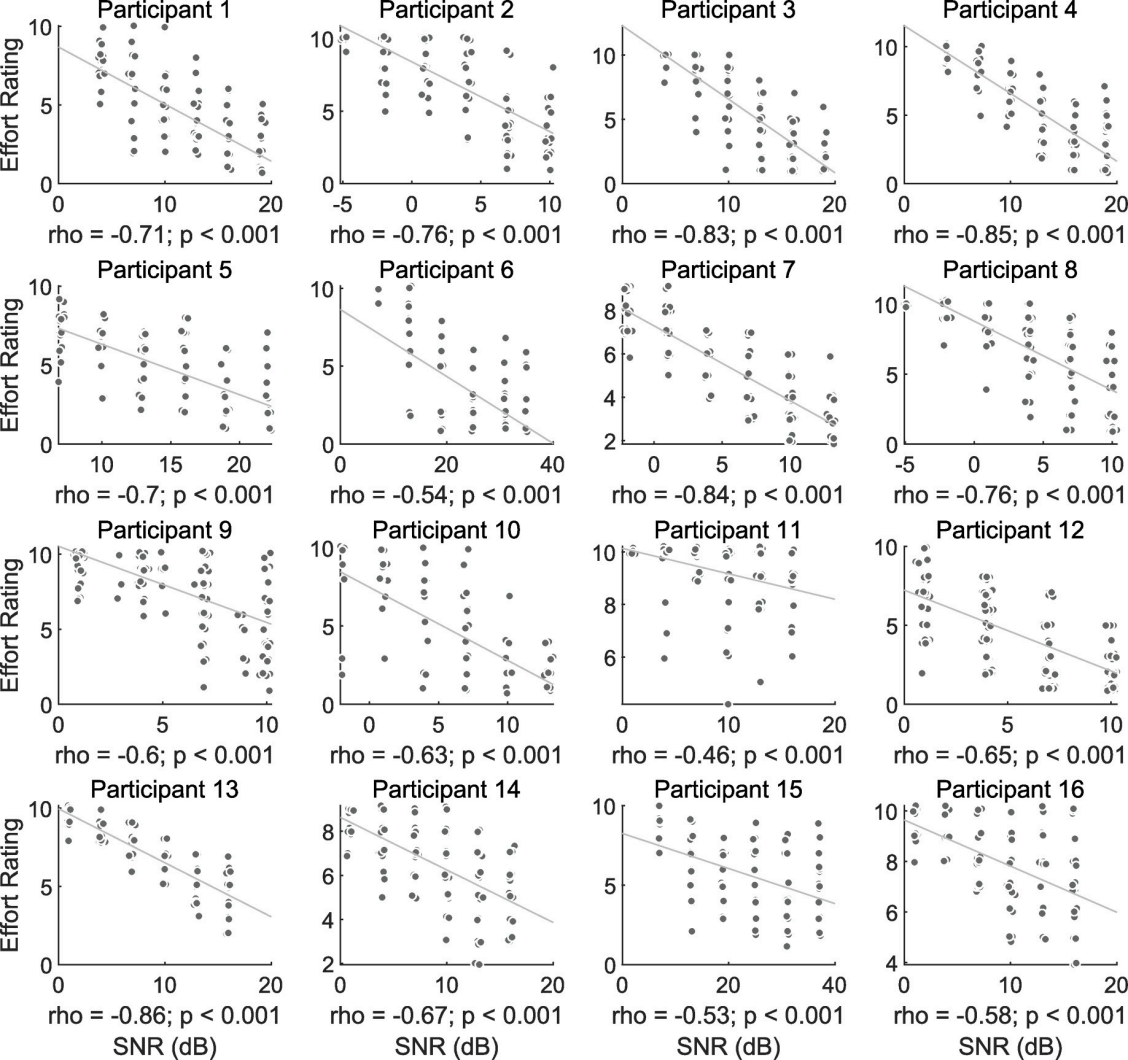
Single-subject effort–SNR correlations. Scatterplots for each participant showing the relationship between the trial SNR and effort ratings. Spearman’s rank correlation coefficients are provided below each plot with significance values. All lines represent the least squares fit. Correlations for all participants were significant, and survived false discovery rate correction. Note that values are jittered around each effort level and SNR level for visibility.

Per individual, we also tested for linear relationships between effort ratings and SNR level. As expected, these variables were significantly related for each CI user (all p values survived false discovery rate correction). As SNR decreased, effort ratings increased. Individual plots are shown in Fig 3.

Next, we conducted a single-trial analysis that examined the effect of trial SNR and effort ratings on task performance using a logistic linear mixed effects model. The results indicated a main effect of effort (OR = 0.150, SE = 0.043, z = −6.86, p < 0.001) and a main effect of SNR (OR = 2.10, SE = 0.571, z = 2.72, p = 0.007). The results suggest that not only SNR, but self-reported effort explained variability in task performance. Prediction curves and 95% confidence intervals for each term are plotted in Fig 2B and 2C. Performance predicted by the model tends to decline with higher effort ratings, especially above effort ratings at and above 5. Predicted performance also tends to decrease with decreasing SNR, which is sensible considering that lower SNR trials have higher noise levels that likely to lead to incorrect responses.

### Parietal alpha power as a function of listening effort

**Fig 4:**
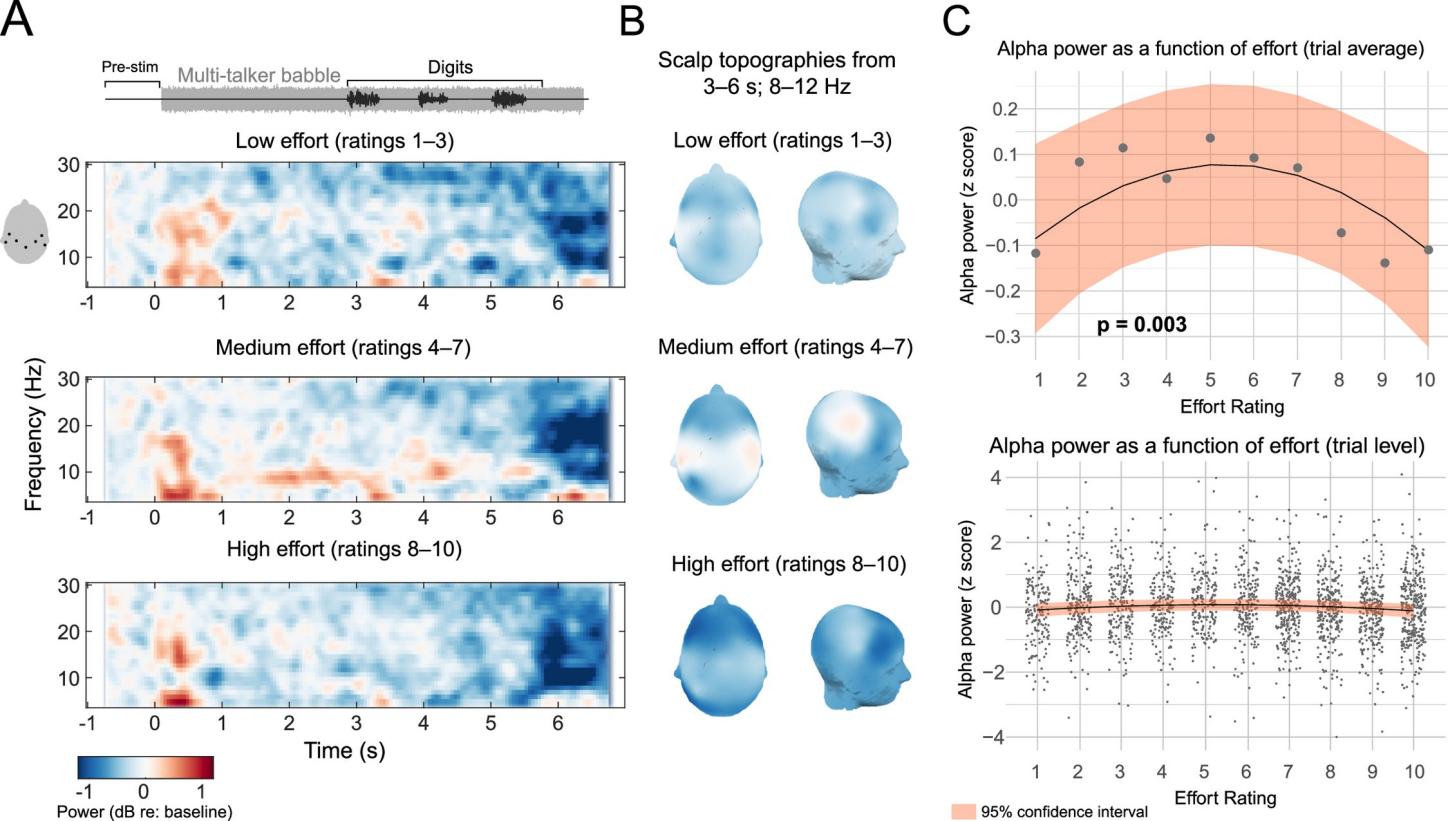
Parietal EEG alpha power as a function of listening effort in CI users. A) The three time-frequency plots depict wideband power (expressed as a dB change relative to baseline) averaged across low, medium, and high effort rating groupings in seven posterior channels (channel locations plotted adjacent to the top plot). These bins, though unbalanced between categories, were chosen for the sake of illustration. Red shading indicates higher power compared to baseline, and blue shading represents lower power. Above the time-frequency plots is a schematic describing the acoustic waveform across the trial structure, including the babble noise and digits. Note that effort groupings are for visualization, as single trials were the basis for inferential analysis. Further, these plots are an average of time frequency representations across participants, where trials were averaged before time frequency decomposition. Single-trial time frequency representations were submitted to mixed modeling. B) Topographic maps showing alpha power averaged over the 3- to 6- second time window. The left column is a top view of the scalp, and the right column is the right view of the scalp. C) The upper panel plots the inverted U function between listening effort ratings and alpha power (z-scored). The grey dots represent averaged alpha power at each effort rating level. The black line and orange shaded area are the regression lines from the single-trial analysis, bounded by 95% confidence intervals. The lower panel plots the same regression line and confidence intervals as in the upper panel, but the grey dots are z-scored alpha power for each trial. Note that individual trials are jittered around each effort level for visibility.

Fig 4A depicts time-frequency plots of seven parietal sensors chosen for analysis. For purposes of visualization, oscillatory power expressed as a dB change from baseline is shown across parietal sensors and averaged across three effort “groupings” for low, medium, and high ratings. Alpha power as a dB change from baseline (herein, simply alpha power) appears to be comparatively higher in magnitude for medium effort ratings, and comparatively lower in magnitude for medium and higher effort ratings. Fig 4B shows topographical maps for alpha power during the digit presentation period (3–6 s) for each of the effort groupings, with alpha power being highest for the medium effort grouping and lowest for the high and low grouping. These qualitative observations are suggestive of an inverted U-shape function. Note that low, medium and high groupings were not used in the analysis and that bins are unbalanced, but are presented for the sake of illustration. No inferences are drawn based on these bins.

Mixed effects modeling on single trials agreed with the qualitative depiction in Fig 4A, and indicated that the quadratic term for effort was significant (standardized β = –0.073, SE = 0.0265, F(1,2205.3) = 7.66, p = 0.006). No other fixed effects reached significance (p’s > 0.29). For purposes of visualization, regression lines and the 95% confidence interval for the quadratic relationship are plotted in Fig 4C, upper panel against the average power value (z-scored) per effort rating level across the 16 participants. The lower panel of Fig 4C shows the model fit against z-scored alpha power in individual trials. The inverted U shape suggests that effort is associated with relatively higher alpha power in parietal scalp regions when effort is at the midpoint of the scale, and is lower when effort ratings are either higher or lower.

An open question for physiological measures of effort is if the inverted U-shaped function reflects the process of “giving up” [51,54]. That is, the downturn in alpha power (or any physiological measure) from the midpoint of the effort rating scale as ratings increase toward the measurement ceiling may signal that participants are no longer exerting effort due to the difficulty of the trial. This would also assume that alpha power scales linearly and positively with subjective effort ratings, and moreover, that participants were rating the perceived difficulty of the trial and not the effort expended, in contradiction to the instructions given by the experimenter. To test assumption, we ran a second mixed effects regression model on trials on which participants correctly reported all three digits. The coefficient for the quadratic effort term was nearly identical to the model without omission of these trials, and remained significant (standardized β = –0.074, SE = 0.0310, F(1534.4) = 6.76, p = 0.017). No other fixed effects terms were significant (p’s > 0.64). This result is inconsistent with the notion that the downward slope of the inverted U shape reflects participants’ disengagement from the task at high effort ratings, because it is unreasonable to assume that participants who “gave up” were able to correctly report all digits in the correct order.

### Left IFG alpha power as a function of listening effort

**Fig 5:**
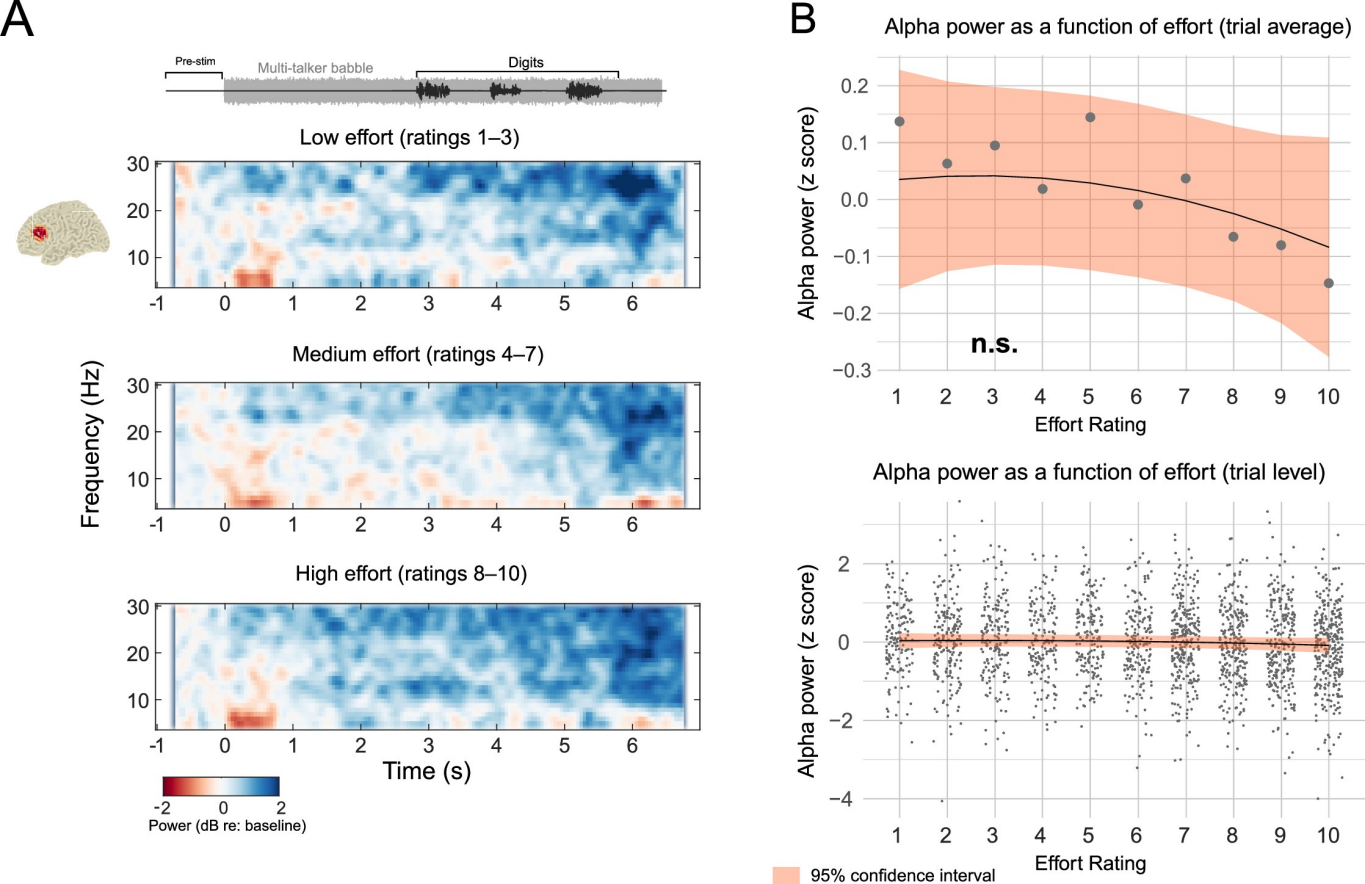
Left IFG alpha power as a function of listening effort in CI users. A) Same as Fig 4A; the location of left IFG is shown adjacent to the top time-frequency plot. B) The upper panel plots the function between listening effort ratings and alpha power. The grey dots represent an average of z-scored alpha power values per each effort rating level. The black line and orange shaded area are the regression lines from the single-trial analysis, bounded by 95% confidence intervals. The lower panel is the same as the upper panel, but the grey dots represent z-scored alpha power values for individual trials. Note that the regression line and confidence intervals are identical between the upper and lower panels. Note that individual trials are jittered around each effort level for visibility. n.s. = not significant.

A similar analysis was performed on EEG data that were spatially filtered to emphasize sources in the left inferior frontal gyrus. Alpha power in left IFG has been linked to subjective effort ratings in a comparable task design [35]. Fig 5A shows time-frequency plots sourced to left IFG for effort grouped as low, medium, and high. Alpha power from 8 to 12 Hz does not appear to modulate across the trials in the low and medium effort rating groupings. Mixed effects modeling found that no fixed effects terms reached significance (p’s > 0.083), including the linear term for effort (p = 0.19). Fig 5B shows that alpha power (z-scored) tended to decline as effort increases, but this trend was not significant. Fig 5B plots z-scored single-trial IFG alpha power as a function of effort. These results do not support the view that subjective effort ratings relate to alpha power in left IFG within this task design. We note that the data processing steps between parietal sensors and left IFG are fundamentally different, and the models for these two regions should not be directly compared.

## Discussion

### Summary

In a sample of CI users, we tested for a relationship between subjective reports of listening effort level in a speech-in-noise task and the power of EEG cortical alpha oscillations for two candidate locations, a source estimate of left inferior frontal gyrus, and sensors over parietal cortex. Consistent with our hypothesis for parietal sensors, we found an inverted U-shaped function between effort ratings and alpha power in parietal sensors, but evidence of a relationship in left IFG was equivocal and the hypothesis for this area was not supported. We importantly highlight that the analyses for parietal sensors and source estimates of left IFG are separate and are not directly compared, and we do not conclude that alpha power tracks effort *only* in parietal sensors. We chose these analysis methods as they represent two measures of alpha power that have been directly compared to subjective effort ratings in prior studies [32,35].

Behavioral results indicated that across CI users, the average level of performance in the task corresponded to clinical scores used to measure speech perception. From single-trial behavioral analysis, effort negatively correlated with performance, and trial SNR was positively correlated with performance. Results are discussed in terms of hypothesized functions for cortical alpha oscillations, and potential clinical implementation.

### The relationship between subjective listening effort and parietal alpha power

Several studies have reported nonlinear relationships between candidate measures of listening effort and the conditions of difficult listening tasks. For instance, inverted U-shaped functions emerge as a function of decreasing SNR (for pupillometry [52,55]; for effort ratings and reaction times on dual-task designs, [51]) or for decreasing speech understanding (for parietal alpha power, [34]). The inverted U shape has been taken as evidence of “cognitive overload,” where reaction times and pupil diameter increase with task difficulty, but as the listening conditions become more difficult, the participant disengages from the task and effort measures decrease [54].

The inverted U-shaped function in the present study was not between task SNR and alpha power, but rather described changes in alpha power as a function of effort ratings. Critically, this relationship was maintained when trials were removed in which the participants responded incorrectly. The preservation of the inverted U shape function is inconsistent with the notion of participant disengagement, as it is unlikely that a participant was “giving up” yet able to report the majority of digits in correct order. As a result, we can rule out participant disengagement as an interpretation for the inverted U function observed in this study.

Nonetheless, our confirmatory finding is in contradiction to prior reports that directly or indirectly compared effort ratings and alpha power. In normal-hearing young adults, McMahon et al. [32] for instance found effort ratings decreased with speech in noise that was additionally spectrally degraded, but the same relationship was not found for parietal alpha power. Also using normal-hearing participants, Decruy et al. [34] found an inverted U shape function between parietal alpha power and the level of speech in noise understanding (more power in medium levels of understanding, but less power at high and low understanding), but effort ratings did not show this relationship. Alhanbali et al. [21] used a similar digit triplet in noise test for a mixed sample of individuals with hearing loss and normal hearing. They found that parietal alpha power did not correlate with effort ratings as measured by the NASA-TLX, but did find a weak negative relationship with self-reported fatigue on a visual analogue scale. Finally, Wöstmann et al. [27] found that modulations of alpha power during speech-in-noise listening were associated with a listener’s overall perception of the difficult when listening in noise in everyday life; however these ratings were not reflective of the effort given in the task itself. In our study, the U-shaped relationship may have been uncovered due to our experimental design that fit each individual’s effort span to a range of SNRs, which differs markedly from designs that aimed for one target performance level or multiple levels of speech understanding [21,34,35].

The functional meaning of an inverted U pattern for alpha power values with increasing effort is unclear, as a mechanistic explanation for parietal alpha oscillations during difficult listening tasks is not firmly established. As discussed, the “functional inhibition” hypothesis describes increased alpha power as consequence of inhibition in neural circuits, and decreased alpha power reflects neural engagement by reducing suppression [29,30]. Across studies, there have been observations of both an increase in in alpha power with speech listening difficulty (e.g., [27,28,32,56]) as well as decreases [33,57]. The difference in directions between these studies may be due to task-based differences or variability in listener strategies to complete the task, in line with the view that alpha power in auditory experiments changes as a function of the goals of the listeners, including selection of task-relevant stimuli [31,58,59]. The suggestion is that parietal alpha power may reflect supramodal stimulus processing within a dorsal attention network [60], with higher alpha power indicating inhibition of modal representations (e.g., visual input) that are not necessary for the goals of the task.

Contextualizing the present data within this framework, increases in alpha power as effort ratings increase (as in, from low to medium effort) can be taken as the application of cognitive resources toward inhibition in attention networks that may serve to “filter out” competing sensory inputs or perhaps the babble noise itself (increased alpha). However, as effort increases further toward high levels, a provisional interpretation is that the downturn of the U shape could indicate a reversal of the sensory gating mechanism, where nonauditory sensory input is no longer suppressed but rather leveraged to assist speech perception (decreased alpha). At high effort levels, for instance, it may be reflexive under these conditions for multisensory networks to use rather filter out visual information (e.g., potential availability of mouth or face movements) or other sensory information to facilitate the formation of speech objects [61–63]. A caveat is that the inverted U shape was observed despite the fact that informative visual or sensory cues were not available to the listener. The alpha decrease could thus be interpreted as the release of a general sensory gating mechanism in anticipation of potentially useful sensory cues in the environment that can aid the listening-in-noise task. This speculation requires further testing.

An alternative interpretation is that the upward and downward trajectories of alpha represent spatially overlapping but distinct processes [59] arising from subcomponents of the parietal attention network or in dorsal and ventral streams [64]. For instance, there may be inhibitory operations as discussed [29], but decreases in alpha power at high effort levels may reflect a response in parietal attention networks to task demands or a functionally separate process involved in the enhancement of target speech [59] A recent report, for instance, found that alpha power-related target enhancement was functionally separable from distractor suppression in a spatial pitch discrimination task [65]. With increasing subjectively rated effort, the decrease in alpha power relative to baseline observed in this study may reflect an enhancement of the target speech that is separate from the alpha power modulations related to a suppression of the background babble noise.

The role of working memory must also be considered when interpreting the relationship between alpha power and effort ratings. Although the current was not designed to test working memory, working memory is clearly involved in speech-in-noise processing [10], and may have been implicated here when participants stored presented digits for recall during the reporting period of the trial. Alpha power has been reported to both increase and decrease with more load, but for verbal working memory, more studies tend to report an increase in alpha power rather than a decrease [66]. Results from Petersen et al [56] may be more informative, where the authors found that the relationship between speech memory load, background noise, and degree of hearing loss is complex. They found that alpha power increases with higher memory load and background noise in typical-hearing and mild-hearing-loss listeners, but alpha power “breaks down” and is lower for listeners with moderate hearing loss. These latter listeners may have reached the limit of available cognitive resources for working memory, explaining the downturn of alpha power. Inspired by this view, the current data could be interpreted in light of cognitive resources that are devoted to both speech processing and storage of verbal information in working memory. As effort increases, cognitive resources may be devoted to storing the digit representations in working memory as indexed by an increase in alpha oscillations. But as effort increases further due to the demands of listening, resources may be rerouted to processes the sensory representations (i.e., target selection) at the cost of fewer resources directed to information storage. It would be interesting for future studies to manipulate memory load to see how alpha power varies as a function of listening effort.

### The relationship between subjective listening effort and left IFG alpha power

Inconsistent with the results of Dimitrijevic et al. [35], we found that the relationship between alpha oscillations and effort ratings in left IFG was not significant. Although the task was similar between the two studies, several differences between the designs and signal processing may account for the discrepancy. Implementation of the common spatial filter, for example was done across all trials (thus SNRs) presently, whereas in Dimitrijevic et al. [35], the spatial filter was constructed based on presentation of speech in noise at a fixed performance level of 50%. This procedure may change filter weights appreciably.

With respect to experimental design, the positive correlation in Dimitrijevic et al. [35] described differences in effort ratings and alpha power *between* subjects, while the current design tested this relationship within subjects. Second, performance on each trial was fixed in the former study at a speech reception threshold of 50%, while here, the SNR varied from trial to trial (SRT approximated 71% in the current study), and was determined through a pre-experiment psychophysical task to identify SNR ranges suitable for a range of low (one) and high (ten) effort. Effort ratings in Dimitrijevic et al. [35] tended to be higher, spanning from five to ten. Finally, stimuli in the current study were delivered through a speaker positioned in front of the participant while babble noise was in the peripheral speakers, and in Dimitrijevic et al., [35] the noise and talker were co-located to one speaker in front of the participant. It is thus plausible that noise masking differences and conditions in which speech-in-noise were tested influenced the way that listeners perceived and responded to the stimuli, producing differences in patterns of neural oscillations between the two studies.

### Implications for understanding listening effort in clinical populations

Behavioral performance on the digits-in-noise task positively correlated to the CNC word recognition score, a standard clinical speech assessment test used to describe CI outcomes. We interpret this finding to indicate that the experimental task in part reflected the clinical status of CI users’ speech perception ability, and thus demonstrates a potential clinical utility for our stimulation paradigm that permits concurrent monitoring of brain activity using EEG. Furthermore, we found that effort was a significant predictor of CI users’ performance on the digits-in-noise task in addition to trial SNR. These findings indicate that subjective effort, not just task difficulty, factors into speech-in-noise listening ability and thus needs to be considered in clinical evaluation.

An open question is if alpha oscillation measurement can be leveraged to yield a reliable neural marker of listening effort [5,8,21]. U-shaped results of the parietal sensor analysis suggest that a conditional interpretation of alpha power would be needed in a clinical setting. For instance, under highly demanding listening conditions, lower parietal alpha power may signal higher effort. Conversely, under less demanding listening contexts, higher parietal alpha power may indicate higher effort. These assumptions require further testing.

### Limitations and future directions

There are limitations to the current study, each of which open future research directions. First, the three-digit speech stimuli in this study are different from continuous, natural speech encountered in realistic listening environments. We aimed to remove the influence of linguistic factors (e.g., contextual cues such as semantic meaning which are known to facilitate speech in noise perception [67]) and are known to modulate activity in left IFG [37] so that the role of alpha oscillations in noisy listening situations was better isolated from linguistic ability. Nonetheless, linguistic factors are an irrefutable part of daily listening experience, and for a physiological effort measure to be useful, it should be robust in ecologically valid listening scenarios. Future studies should replicate this design, but use complete sentences or continuous, natural speech.

Second, this study did not investigate the relationship between speech encoding and self-reported effort ratings. Dimitrijevic et al. [35] reported that individuals with higher effort ratings had poorer cortico-acoustic coherence (i.e., neural tracking) between auditory cortex and the acoustic speech signal at oscillatory frequencies between 2–5 Hz. We could not obtain similar measures of here because the number of trials per SNR was too low (≤ 30 in each SNR condition) for such metrics. Future experiments should devise experimental protocols that allow an examination of alpha power changes and speech tracking on a within-subject level.

Third, our analysis did not plan for or analyze the time course of alpha power across the trial. Inspection of the time-frequency plots for both parietal sensors and left IFG (Figs 4A and 5A) suggests that alpha power modulates, which might reflect listening effort, before the onset of the digits. We chose the 3- to 6-second time window *a priori* to be consistent with Dimitrijevic et al. [35], but future studies should be designed to be sensitive to the temporal characteristics of alpha power and how it relates to subjective effort reports.

Finally, the goal of the study was to test for relationships between effort ratings and cortical alpha oscillations at two different locations using methods that were similar to processing steps used in prior studies. This was done to afford a closer comparison to past work using our novel method. However due to the mixture of source and sensor activity due to past methods, it is difficult to directly compare the findings. More specifically, it is difficult to select sensors that may directly represent left IFG generators, while in turn, generators of parietal alpha power changes are not yet fully established and ROIs are not simply selected. We note that differences in significance between the two models (left IFG and parietal areas) do not mean that two results are significantly different [68], and we do not conclude that source alpha power in left IFG and sensor power in posterior electrodes reflect different processes nor do we conclude that parietal alpha power tracks alpha power better than left IFG.

## Conclusions

Individuals with hearing loss often feel that they must exert a high amount of effort to understand speech in a noisy environment. In this study, we found that self-reported effort was reflected in 8–12 Hz (alpha) oscillations in parietal scalp sensors, when CI users were performing a speech-in-noise task. Importantly, we demonstrated that these results are unlikely to reflect the process of participants “giving up” as the task gets harder. Our findings overall contribute to the development of objective neural markers of listening effort that are intended for clinical use.

## Supporting information

S1 Table Outputs from statistical models. Estimates = slope coefficients; std. error = standard error; Sum sq = sum of squares, DF = degrees of freedom.
(DOCX)
