# Supplementary material for: Cortical alpha oscillations in cochlear implant users reflect subjective listening effort during speech-in-noise perception
Source: PLoS One. 2021 Jul 9;16(7):e0254162. doi: 10.1371/journal.pone.0254162 (PMC8270138; doi:10.1371/journal.pone.0254162)
Supplement: S1 Table — Estimates = slope coefficients; std. error = standard error; Sum sq = sum of squares, DF = degrees of freedom. (DOCX) [file pone.0254162.s001.docx]

**Supporting information**

**S1 Table.**

**Mixed effects binomial logistic regression for single-trial behaviour**

|  | **Odds Ratio** | **Std. Error** | **Z-value** | **P-value** |
| --- | --- | --- | --- | --- |
| **Effort** | 0.151 | 0.0408 | -6.98 | 2.90E-12 |
| **SNR** | 2.10 | 0.571 | 2.71 | 0.0066 |

**Mixed effects model for single-trial parietal alpha power (all trials)**

|  | **Estimate** | **Std. Error** | **Sum Sq** | **DF** | **F-value** | **P-Value** |
| --- | --- | --- | --- | --- | --- | --- |
| **Effort** | -0.0362 | 0.0339 | 1.0221 | 1, 1725.6 | 1.1402 | 0.2858 |
| **Effort^2** | -0.0733 | 0.0265 | 6.8654 | 1,2205.3 | 7.6586 | 0.0057 |
| **SNR** | 0.0252 | 0.0436 | 0.2998 | 1, 1346.5 | 0.3344 | 0.5631 |
| **SNR^2** | -0.0004 | 0.0158 | 0.0007 | 1, 2143.6 | 0.0007 | 0.9784 |

**Mixed effects model for single-trial parietal alpha power (correct trials only)**

|  | **Estimate** | **Std. Error** | **Sum Sq** | **DF** | **F-value** | **P-Value** |
| --- | --- | --- | --- | --- | --- | --- |
| **Effort** | -0.0048 | 0.0377 | 0.0142 | 1, 1090.8 | 0.0162 | 0.8988 |
| **Effort^2^** | -0.0744 | 0.0310 | 5.0611 | 1, 1534.4 | 5.7566 | 0.0166 |
| **SNR** | 0.0094 | 0.0489 | 0.0323 | 1, 848.0 | 0.0367 | 0.8480 |
| **SNR^2^** | 0.0086 | 0.0183 | 0.1934 | 1, 1466.5 | 0.2199 | 0.6392 |

|  | **Estimate** | **Std. Error** | **Sum Sq** | **DF** | **F-value** | **P-Value** |
| --- | --- | --- | --- | --- | --- | --- |
| **Effort** | -0.0472 | 0.0777 | 1.6305 | 1, 1189.29 | 1.7541 | 0.1856 |
| **Effort^2^** | -0.0196 | 0.0277 | 0.4645 | 1, 1997.53 | 0.4997 | 0.4797 |
| **SNR** | 0.0793 | 0.0457 | 2.8032 | 1, 776.82 | 3.0156 | 0.0829 |
| **SNR^2^** | -0.0086 | 0.0167 | 0.2428 | 1, 1864.78 | 0.2612 | 0.6093 |

**Mixed effects model for single-trial left IFG alpha power**

**S1 Table.** **Outputs from statistical models.** Estimates = slope coefficients; std. error = standard error; Sum sq = sum of squares, DF = degrees of freedom.
